# Supplementary material for: Risk of psychiatric disorders among the surviving twins after a co-twin loss
Source: eLife. 2020 Jul 14;9:e56860. doi: 10.7554/eLife.56860 (PMC7360364; doi:10.7554/eLife.56860)
Supplement: Supplementary file 1. — Supplementary Table 1. International Classification of Diseases (ICD) codes, eighth (ICD-8; 1969–1986), ninth (ICD-9; 1987–1996), and tenth (ICD-10; 1997–2013) Swedish revisions for diagnoses used in this study Supplementary Table 2. Hazard ratios (HRs) with 95% confidence intervals (CIs) for any psychiatric disorder among the surviving twins after co-twin loss, compared to matched unexposed twins or their full siblings, by different characteristics Supplementary Table 3. Hazard ratios (HRs) with 95% confidence intervals (CIs) for any psychiatric disorder, identified by only primary diagnosis in the National Patient Register or by only diagnosis from National Inpatient Register, among the surviving twins after co-twin loss, compared to matched unexposed twins (matched twin cohort) or their full siblings (twin-sibling cohort). [file elife-56860-supp1.docx]

Supplementary Table 1. International Classification of Diseases (ICD) codes, eighth (ICD-8; 1969–1986), ninth (ICD-9; 1987–1996), and tenth (ICD-10; 1997–2013) Swedish revisions for diagnoses used in this study

|  | Specific subtypes | **ICD-8** | **ICD-9** | **ICD-10** |
| --- | --- | --- | --- | --- |
| **Cause of death** |  |  |  |  |
| Unnatural death: death with any external cause |  | E807-E999 | E807-E999 | V, X, Y, W |
| **Outcomes** **identification** | |  |  |  |
| All psychiatric disorders |  | 290–315 | 290–315 | F |
| Subtypes of psychiatric disorders | Mood disorders, *mainly including depressive disorder and bipolar disorder* | 296, 300.4 | 296, 300E, 311 | F30-F39 |
|  | Anxiety disorders | 300.0, 300.2 | 300A, 300C | F40-F41 |
|  | Stress-related disorder | 307, 308.4 | 308, 309 | F43 |
|  | Substance misuse | 291, 303, 304 | 291, 303, 304, 305A, 305X | F10-F19 |
|  | Psychotic disorder | 295, 297, 298, 299 | 295, 297, 298, | F20-F29 |
| **Covariates** |  |  |  |  |
| History of severe somatic conditions | Myocardial infarction | 410 | 410,412 | I21, I22, I25.2 |
|  | Congestive heart failure | 427.00-427.27, 428.99 | 428 | I50 |
|  | Cerebrovascular disease | 430-438 | 430-438 | G45, G46, I60-I69 |
|  | Chronic pulmonary disease | 460-466, 490-493, 502-508 | 490-496 | J40-J47 |
|  | Connective tissue disease | 710-718 | 710A, 710B, 710E, 714A, 714B, 714C, 714W,714X, 725 | M05, M06, M32-M34, M35.1, M35.3 |
|  | Diabetes | 250.00 | 250 | E10-E14 |
|  | Renal diseases | 580-584, 590 | 582,583 | N01, N03, N05.2-N05.7 |
|  | Liver diseases | 570-576 | 571C, 571E,571F, 571G, 572C, 572D, 572E, 572W, 456A, 456B, 456C | K70.2-K70.4, K71.7, K72.1, K72.9, K73, K74, K76.6, K76.7 |
|  | Ulcer diseases | 530.93, 531-534 | 531-534 | K25-K28 |
|  | HIV infection/AIDS | - | 042-044 | B20-B24 |

**Supplementary Table 2. Hazard ratios (HRs) with 95% confidence intervals (CIs) for any psychiatric disorder among the surviving twins after co-twin loss, compared to matched unexposed twins or their full siblings, by different characteristics**

|  | **Matched twin cohort: twins** exposed vs. unexposed to loss of a co-twin | | **Twin-sibling cohort: twins** exposed to loss of a co-twin vs. **their full siblings** | |
| --- | --- | --- | --- | --- |
|  | Number of cases (Crude incidence rate, per 1000 person years), exposed / unexposed twins | HR (95% CI)^*^ | Number of cases (Crude incidence rate, per 1000 person years), exposed twins/ exposed siblings | HR (95% CI) ^*^ |
| *By sex* |  |  |  |  |
| Male | 276(12.69)/783(6.93) | 1.68 (1.46-1.94) | 176(12.43)/201(7.72) | 1.52 (1.13-2.04) |
| Female | 250(11.87)/738(6.87) | 1.61 (1.39-1.87) | 152(11.37)/214(8.08) | 1.25 (0.91-1.72) |
| *By age at the index date, years* |  |  |  |  |
| 2-18 | 51(12.92)/144(6.51) | 2.08 (1.47-2.95) | 30(11.60)/26(6.56) | 2.23 (0.92-5.41) |
| 19-52 | 208(11.45)/525(5.48) | 1.98 (1.67-2.35) | 144(11.15)/173(6.85) | 1.80 (1.35-2.40) |
| 53-64 | 143(10.83)/461(7.00) | 1.44 (1.18-1.76) | 90(11.15)/129(7.92) | 1.30 (0.88-1.92) |
| ≥ 65 | 124(16.59)/391(10.66) | 1.28 (1.03-1.59) | 64(16.17)/87(12.40) | 1.31 (0.80-2.17) |
| *By family history of psychiatric disorder* |  |  |  |  |
| Yes | 271(15.64)/594(9.27) | 1.56 (1.28-1.89) | 168(15.23)/210(9.48) | 1.61 (1.27-2.03) |
| No | 255(10.01)/927(5.93) | 1.70 (1.45-1.99) | 160(9.70)/205(6.75) | 1.50 (1.19-1.89) |
| *By cause of the co-twin’s death* |  |  |  |  |
| Unnatural death | 170(12.79)/425(6.09) | 2.05 (1.70-2.47) | 106(11.48)/150(8.58) | 1.45 (1.09-1.92) |
| Natural death | 356(12.06)/1096(7.28) | 1.49 (1.32-1.69) | 222(12.14)/265(7.57) | 1.62 (1.32-1.98) |
| *By age difference: compared to the deceased twin^†^, years* |  |  |  |  |
| 0-2 | - | - | 108(11.85)/104(9.79) | 1.19 (0.87-1.62) |
| 3-5 | - | - | 174(11.55)/134(7.32) | 1.54 (1.19-2.00) |
| >5 | - | - | 176(12.59)/177(7.51) | 1.72 (1.35-2.18) |

**^*^** Cox regression models were stratified by matching identifiers (birth year and sex, in matched twin cohort) or family identifiers (in twin-sibling cohort), and adjusted for education level, family income, marital status, history of severe somatic diseases, and family history of psychiatric disorder. Time since the index date was used as underlying time scale.

^†^ The categorization was done according to the age of exposed non-twin full siblings compared to the deceased twin.

Supplementary Table 3. Hazard ratios (HRs) with 95% confidence intervals (CIs) for any psychiatric disorder, identified by only primary diagnosis in the National Patient Register or by only diagnosis from National Inpatient Register, among the surviving twins after co-twin loss, compared to matched unexposed twins (matched twin cohort) or their full siblings (twin-sibling cohort)

|  | Number of cases (Crude incidence rate, per 1000 person-years), exposed / unexposed | HR (95% CI)^*^ |
| --- | --- | --- |
| *Only* ***primary*** *diagnosis in National Patient Register* |  |  |
| Matched twin cohort | 395(9.12)/1081(4.87) | 1.75 (1.55-1.97) |
| Twin-sibling cohort | 247(8.86)/288(5.44) | 1.66 (1.37-2.01) |
| *Only diagnosis from National* ***Inpatient*** *Register* |  |  |
| Matched twin cohort | 320(7.28)/877(3.92) | 1.73 (1.51-1.98) |
| Twin-sibling cohort | 199(7.05)/258(4.84) | 1.51 (1.23-1.86) |

**^*^** Cox regression models were stratified by matching identifiers (birth year and sex, in matched twin loss-twin cohort) or family identifiers (in twin-sibling cohort), and adjusted for education level, family income, marital status, history of severe somatic diseases, and family history of psychiatric disorders. Time since the index date was used as underlying time scale.
